# Repetitive transcranial magnetic stimulation to alleviate fatigue in multiple sclerosis—study protocol for a randomized sham-controlled double-blinded clinical trial

**DOI:** 10.3389/fneur.2026.1759679

**Published:** 2026-02-26

**Authors:** Sofus Nygaard, Mads Alexander Just Madsen, Vanessa Wiggermann, Chiara Cabras, Lasse Christiansen, Alena Svatkova, Henrik Lundell, Helene Højsgaard Chow, Jeppe Romme Christensen, Morten Blinkenberg, Finn Sellebjerg, Hartwig Siebner

**Affiliations:** 1 Danish Research Centre for Magnetic Resonance, Department of Radiology and Nuclear Medicine, Copenhagen University Hospital—Amager and Hvidovre, Hvidovre, Denmark; 2 MS Center Amsterdam, Anatomy and Neurosciences, Amsterdam Neuroscience, Amsterdam University Medical Center, location VUmc, Vrije Universiteit Amsterdam, Amsterdam, Netherlands; 3 Department of Nutrition, Exercise and Sports, University of Copenhagen, Copenhagen, Denmark; 4 Department of Radiology and Nuclear Medicine, Copenhagen University Hospital—Amager and Hvidovre, Hvidovre, Denmark; 5 MR Section, DTU Health Tech, Technical University of Denmark, Lyngby, Denmark; 6 Department of Neurology, Danish Multiple Sclerosis Center, Copenhagen University Hospital—Rigshospitalet, Glostrup, Denmark; 7 Department of Clinical Medicine, Faculty of Health and Medical Sciences, University of Copenhagen, Copenhagen, Denmark; 8 Department of Neurology, Copenhagen University Hospital—Bispebjerg and Frederiksberg, Copenhagen, Denmark

**Keywords:** fatigue, MR spectroscopy, multiple sclerosis, premotor cortex, randomized controlled clinical trial, repetitive transcranial magnetic stimulation, transcranial magenetic stimulation, ultra high field magnetic resonance imaging

## Abstract

**Background:**

Fatigue is a common and disabling symptom in multiple sclerosis (MS), quantifiable by patient reported outcome instruments such as the Fatigue Scale for Motor and Cognitive Functions (FSMC). The pathophysiology of fatigue remains poorly understood, and effective treatments are limited. Emerging evidence implicates disrupted excitation–inhibition balance in the premotor cortex as a potential culprit of fatigue in MS. Converging evidence now show that such network imbalance can be modulated with repetitive transcranial magnetic stimulation (TMS). The efficacy of premotor rTMS retuning excitation-inhibition balance, thus improving MS-related fatigue, has yet to be examined in a clinical trial.

**Methods:**

This randomized, double-blinded, sham-controlled, parallel-group trial investigates the efficacy of premotor TMS in treating fatigue in MS. Fifty-eight patients with MS will receive either active or sham TMS targeting the left dorsal premotor cortex (PMd). On five consecutive days, participants will undergo 30-min sessions using a novel low-frequency (0.72 Hz) paired-pulse repetitive TMS protocol with an interstimulus interval of 33 ms. The primary endpoint is the change in FSMC score 6 days post-intervention. Secondary outcomes include additional fatigue assessments and quantification of regional γ-aminobutyric acid (GABA) and glutamate concentrations of the targeted PMd, via ultra-high-field (7T) magnetic resonance spectroscopy. We hypothesize that active treatment will result in greater fatigue reduction than sham treatment and correlate positively with an increase in regional GABA in the stimulated premotor region. Exploratory endpoints include structural and functional connectivity changes assessed with 7T resonance imaging and motor cortical excitability changes measured with TMS.

**Discussion:**

This study will assess the feasibility and efficacy of a novel low-frequency paired-pulse TMS protocol for fatigue in MS. Repeated neurophysiological measurements of cortical excitation–inhibition balance will yield mechanistic insights and guide future repetitive TMS trials targeting MS-related fatigue.

**Clinical Trial Registration:**

http://www.clinicaltrials.gov, NCT06569550.

## Highlights

- This trial tests premotor repetitive TMS to reduce fatigue in relapsing-remitting multiple sclerosis.
- 58 patients get active or sham low-frequency paired-pulse repetitive TMS over five days.
- The primary outcome is a change in FSMC scores six days after treatment.
- 7T MR spectroscopy and TMS measures will link fatigue relief to excitation–inhibition balance.

## Background and rationale

Multiple sclerosis (MS) is an immune mediated disease targeting the central nervous system (1), with increasing global prevalence and incidence (2). Fatigue is highly prevalent in MS, affecting up to 78–95% (3, 4) of patients and is among the most disabling MS symptoms (5, 6). In people with MS, fatigue is consistently associated with decreased employment status and lower quality of life (4, 7–9).

The pathophysiology of fatigue in MS remains incompletely understood and involves multiple mechanisms (10–12). One hypothesis is that fatigue arises from an imbalance between cortical excitation and inhibition (13). This theory is supported by neurophysiological studies using electroencephalography (EEG) (14), transcranial magnetic stimulation (TMS) (15–18), and functional magnetic resonance imaging (fMRI) (19–21). Notably, individuals with high trait fatigue exhibit task-related hyperactivation of the premotor cortex during a non-fatiguing grip force task (20). Those patients who were capable of further increasing premotor activity following a fatiguing motor task reported lower fatigue in daily life (21). These findings suggest that trait fatigue is associated with an inefficient allocation of premotor cortical resources. Specifically, the disrupted excitation-inhibition balance may result in premature or excessive premotor activation during routine motor tasks, limiting the capacity of the central motor system for further upregulation when demanded. It is worth noting, that the exact mechanism of this disrupted excitation-inhibition balance remains unclarified, as there are studies that show both increased activation and increased inhibition, depending on modality and experimental setup (22, 23).

Pharmacological treatments targeting fatigue in MS have shown limited clinical relevance in the majority of patients (24–26). Physical exercise, in contrast, consistently shows a clinically relevant and positive impact on MS-related fatigue (24, 27, 28). However, exercise-based interventions may not be feasible for all patients, particularly those with advanced disability. Emerging evidence suggests that the beneficial effects of exercise may be mediated, at least in part, by modulating excitability within motor-related networks (16, 29). Currently, no approved therapies specifically target the imbalance in inhibition and excitation within the premotor-motor network.

Repetitive Transcranial Magnetic Stimulation (rTMS) is a non-invasive neuromodulation technique capable of inducing both local and network-wide plasticity in the brain (30–32). Several studies have shown that rTMS targeting the premotor cortex can produce direct and lasting modulatory effects on motor cortical excitability in healthy individuals (33–36). These effects depend critically on the stimulation parameters, including pulse type (e.g., single vs. paired or burst) and temporal patterning (37–39). When applied to the left dorsal premotor cortex (PMd) at an inter-stimulus interval (ISI) of 33 ms, low-frequency (0.7 Hz) paired-pulse rTMS (pp-rTMS) suppresses resting activity in a bilateral premotor-motor cortical network (40). This acute suppressive effect supports the potential of low frequency paired-pulse rTMS as a targeted intervention for network-level excitation-inhibition imbalances, including for potential alleviation of MS-related fatigue.

Side effects of rTMS include mild and transient local discomfort, headaches, and pain at the stimulation site (41, 42). Serious adverse events, including seizures, are exceedingly rare, when appropriate screening and safety protocols are followed (43, 44).

Few studies have directly examined the efficacy of rTMS for treating fatigue in people with MS. To date, only one small, randomized trial (*n* = 11 per group) has specifically assessed rTMS as treatment of MS-related fatigue, targeting either the motor or prefrontal cortex using an H-coil (45). In that study, no significant difference between active and sham treatment were observed, and the primary focus was safety (45). While several studies have reported reductions in fatigue as a secondary outcome (46–49), trials with the primary aim of alleviating fatigue and investigating the underlying mechanisms hereof are missing.

### Choice of sham comparator

Given the absence of an established, efficacious rTMS-based protocol for fatigue in MS, we have adopted a sham-control design for the present trial. Although other non-invasive brain stimulation modalities, such as transcranial direct current stimulation (tDCS) have shown some positive effect on fatigue in persons with MS (50, 51), considerable methodological heterogeneity across tDCS studies precludes its use as a standardized or mechanistically appropriate comparator. Therefore, a sham-controlled design offers the most rigorous and interpretable framework for evaluating the specific neuromodulatory effects of rTMS on fatigue and its neural correlates.

### Objectives

The primary objective of this study is to evaluate the effect of low-frequency paired-pulse rTMS on patient-reported fatigue in persons with MS. Participants will undergo a five-day intervention consisting of daily 30-min sessions of 0.7 Hz paired-pulse rTMS (inter-stimulus interval: 33 ms) targeting the left PMd. The primary hypothesis is that repeated paired-pulse rTMS of the left PMd will reduce fatigue severity, as measured by the change in Fatigue Scale for Motor and Cognitive Functions (FSMC) scores from baseline to 6 days post-intervention, relative to sham.

The secondary objective of this study is to determine whether changes in fatigue are associated with modulation of the excitation–inhibition balance within the motor and premotor cortex, as measured by ultra-high field 7 Tesla magnetic resonance spectroscopy (7T-MRS) assessed γ-aminobutyric acid (GABA) and glutamate (Glu) concentrations. The secondary hypothesis is that repeated paired-pulse rTMS targeting the left PMd will shift the balance toward relatively increased inhibition, and that the magnitude of this shift toward inhibition will correlate with the degree of individual fatigue reduction.

As an exploratory objective, we will investigate whether changes in fatigue, and its potential alleviation, are reflected in alterations in resting-state functional MRI (rs-fMRI) network dynamics. Additional exploratory outcomes include changes in clinical and behavioral measures related to fatigue.

### Trial design

This is a single-center, parallel-group, two-arm, double-blinded, sham-controlled, randomized trial. Participants will be randomly assigned in a 1:1 ratio to either the active intervention or sham control group. The intervention group will receive five sessions of 30-min rTMS targeting the left PMd, across 5 consecutive days while the control group will receive a sham version of the same protocol.

## Methods: participants, interventions and outcomes

### Study setting

All study procedures take place at the Danish Research Centre for Magnetic Resonance (DRCMR) at Copenhagen University Hospital Amager and Hvidovre, Copenhagen, Denmark. Participants are recruited through the outpatient clinic at Danish Multiple Sclerosis Center, Copenhagen University Hospital - Rigshospitalet, Glostrup, in Copenhagen, Denmark, as well as via the Danish Multiple Sclerosis Society (Scleroseforeningen). Recruitment began in August 2024.

### Eligibility criteria

Inclusion criteria: Participants must meet the following criteria to be eligible for the study: (i) Diagnosis of relapsing–remitting or secondary progressive MS, according to the 2017 McDonald criteria (52), (ii) Presence of moderate to severe fatigue, defined as a Fatigue Scale for Motor and Cognitive Functions (FSMC) score ≥ 53, and (iii) Age between 18 and 65 years.

Exclusion criteria: Participants will be excluded if they meet any of the following conditions: (i) Clinical relapse, corticosteroid treatment, or changes in MS-related medication within 3 months prior to inclusion, (ii) Moderate to severe depression, defined as a Major Depression Inventory (MDI) score ≥ 26 (53, 54) or other severe psychiatric, neurological, or somatic comorbidities, (iii) EDSS score (55) above 6.5 that would limit feasibility of 7T MRI, (iv) Contraindications to 7T MRI (56) or TMS (42, 44) in agreement with current safety guidelines (including pregnancy), (v) intake of pharmaceutical agents that may interfere with the neuromodulatory effect of rTMS, including tricyclic antidepressants (TCAs), monoamine oxidase inhibitors (MAOIs), GABAergic agents (benzodiazepines and gabapentin) and central stimulants such as modafinil. For feasibility reasons selective serotonin reuptake inhibitors (SSRIs), serotonin-norepinephrine reuptake inhibitors (SNRIs) are allowed. Use of selective serotonin receptor agonists (e.g., triptans for migraine) are allowed up to 48 h prior to intervention.

### Intervention—rTMS

Participants will receive five sessions of pp-rTMS, delivered once daily on five consecutive days. Each of the 5 pp-rTMS sessions consists of pulse pairs separated by an ISI of 33 ms (equivalent to 30 Hz) repeated at 0.72 Hz, for a total of 1,300 stimulus pairs per session. Each session will last 29 min and 55 s. Intensity of pp-rTMS will be personalized and adjusted to 80% of the individual’s resting motor threshold (RMT). Stimulation intensity will not be adjusted for difference in scalp-cortex distance between M1 and PMd, due to the low average difference in scalp-cortex distance between these 2 areas (57), and secondarily because a prior study investigating this paradigm over PMd did not adjust intensity, while demonstrating a non-linear relationship between stimulation intensity (40). Individual RMT will be determined on the first intervention day by administering single-pulse TMS over the left primary motor hand area (58). Should the paired-pulse stimulation of left PMd at 80% of RMT produce visible muscle twitches in the right hand, intensity will be reduced until twitches are no longer elicited, to reduce potential participant discomfort during the intervention protocol.

Stimulation will be delivered using a B65-Cool-A/P coil connected to a MagPro XP Orange Edition stimulator (MagVenture, Farum, Denmark). The B65-Cool-A/P coil is a butterfly-shaped coil with active cooling which can be used for both active and placebo stimulation. The MagPro XP Orange Edition stimulator can deliver high-frequency biphasic stimuli trains (2–5 pulses) up to 1,000 Hz with minimal intensity drop-off. To mimic the cutaneous sensation associated with active TMS pulses and preserve blinding, all participants will receive low-intensity electrical stimulation to the forehead, as described in the section on blinding.

Frameless stereotaxy will enable personalized neuronavigated PMd stimulation. The TMS coil position will be continuously controlled with a Localite neuronavigation system based on individual T1-weighted MRI brain scans (Localite, Bonn, Germany). The position of the coil for the duration of the intervention will be ensured with a robotic arm (TMS Cobot, Axilum, Schiltigheim, France), which has been shown to be superior to a rigid coil-holder in maintaining electric field (e-field) at target site during a 30-min session (59).

The stimulation target, the left PMd, is defined in montreal neurological institute (MNI) space (60) at x, y, z-coordinates (34, −2, 66), based on prior findings (21). This target is transformed into subject-specific space using individual T1-weighted and T2-weighted MRI brain scans. The optimal coil position will be calculated with SIMNIBS software (version 4.5^1^) as the scalp position inducing the largest mean electric field in the target region (61). Individual T1w and T2w MRI images will be segmented to a SIMNIBS compatible mesh using CHARM (62). The induced e-field for the B65-Cool coil (63) will be calculated (64, 65) in a 20 mm search radius at 1 mm resolution, with 40-50-degrees of rotation from the mid-sagittal plane, with an angular resolution of 1 degree.

### Intervention—sham rTMS

Participants in the sham group will receive the same low-frequency pp-rTMS protocol as the active group. However, the stimulation coil will be flipped, with the placebo side facing the scalp. This orientation, achieved by rotating the coil along its longitudinal axis, prevents the delivery of a biologically effective magnetic field while preserving the auditory and tactile experience of stimulation. As with the active group, concurrent low-intensity electrical stimulation will be applied near the stimulation site to mimic cutaneous sensations. While this approach reflects current best practice in the field it is worth noting that transcutaneous stimulation may by itself be As recently shown, sham rTMS by transcutaneous electrical stimulation may by itself be considered an active control, rather than an inert control (66).

### Intervention—modifications

The intervention protocol is fixed and cannot be altered at the request of participants. No changes in intensity, session count, or scheduling will be allowed. Modifications are only permitted if visible motor twitches occur, in which case the stimulation intensity will be reduced, as described above.

### Intervention—concomitant care

No additional interventions or medications are restricted during the trial, except as defined in the eligibility criteria. Participants in both study arms may continue their standard care without modification.

### Criteria for discontinuation

Participants may be withdrawn from the study at the discretion of the investigators under the following circumstances: (i) Inability to complete the baseline MRI scan, (ii) occurrence of a clinical MS relapse, (iii) occurrence of a major adverse event or emergence of any new condition contraindicative to rTMS or MRI, and (iv) inability to comply with the intervention schedule or procedures.

All participants retain the right to withdraw from the study at any time, without providing a reason. Participants who have received at least one rTMS session will be included in the intention-to-treat analysis, provided data are available.

### Strategies to improve adherence and participant retention

Participants, who have attended at least one rTMS intervention session, are encouraged to attend the follow-up visit regardless of the amount of intervention sessions completed. Participants who cancel with short notice will be contacted by phone and encouraged to adhere to the intervention. Should participants decide against participation in all intervention days, after initialising the intervention, they will be encouraged to attend as many sessions as they can. This is not considered as a modification of the protocol, but a reduced adherence to the full protocol.

### Primary outcome

The primary outcome of fatigue is measured as the difference in change in total FSMC (67, 68) score from baseline to follow-up at 6 days after the last rTMS intervention, between active and sham stimulation. Rating fatigue is largely subjective, and thus generally assessed as a patient-reported outcome (69, 70). However, the FSMC has the highest construct validity among often-used scales (69).

### Secondary outcome

#### 7 tesla proton magnetic resonance spectroscopy (^1^H-MRS)

The main secondary outcome is the between-group difference in changes in regional neurometabolite concentrations, specifically GABA and Glu, in the targeted left PMd, from baseline to the short-term follow-up on the final day of the intervention (Day 5). These concentrations will be assessed using ^1^H-MRS, acquired from a voxel positioned to cover the left PMd/M1 region. To assess neurometabolic network-level effects, an additional ^1^H-MRS voxel will be acquired in the contralateral (right) PMd/M1 region, to evaluate remote transhemispheric modulation. A voxel placed in the midline parietal region, will serve as a control site not expected to respond to the intervention. In addition to the short-term (Day 5) effects, longer-term effects will also be assessed at the primary follow-up 6 days post-intervention (Day 11), together with clinical outcome measures.

#### Fatigue and fatigability

Complementing the primary endpoint, between-group change in FSMC at 6 days post-intervention, we acquire additional secondary outcomes to assess both subjective fatigue and fatigability. Additional outcomes of subjective fatigue will be measured using the FSMC, at two additional follow-up timepoints: 1 day post-intervention (Day 6) and 4 weeks post-intervention (Day 33).

Objective fatigability will be assessed using the Fatigability Index (FI) (71–73), comparing the change from baseline (Day 0) to the primary follow-up (Day 11). Additionally, patient-reported fatigability will be evaluated using the Pittsburgh Fatigability Scale (PFS) (74, 75), with changes from baseline compared across three follow-up timepoints: Day 6, Day 11, and Day 33.

### Exploratory and other outcomes

#### Fatigue

Each subscale of the FSMC, cognitive and physical, will be analyzed separately as tertiary outcome measures testing for changes during the three follow-up timepoints relative to baseline. Acute fatigue will be quantified immediately before and after each rTMS session with a Visual-Analogue Fatigue Scale (76) assessing between-group differences in session-wise changes. Participants will also complete a fatigue diary during the intervals from baseline (D0) to the first intervention session (Day 1), and from the final session (Day 5) to the primary follow-up (Day 11). As no validated MS-specific fatigue diary currently exists, we will use a customized version adapted from a validated tool in oncology populations (77). Between-group differences in change in average daily fatigue scores will be assessed. To facilitate cross-study comparisons, participants will additionally complete the Fatigue Severity Scale (FSS) (78, 79) and the Modified Fatigue Impact Scale (MFIS) (80–82). These scales will be collected for reference purposes.

#### Depression, quality of life, and sleep

Depression will be assessed at baseline and the primary follow-up (Day 11) using the Major Depression Inventory (MDI) (53, 54), as depression is a key confounder due to its correlation with fatigue (83). Complementing the MDI, the Beck Depression Inventory-II (BDI-II) (84) will also be administered at both timepoints for cross-study comparability, and BDI-II data will only be reported descriptively.

The Multiple Sclerosis Impact Scale (MSIS-29) (85), and the Epworth Sleepiness Scale (ESS) (86) will be acquired at four timepoints [baseline; 1 day post-intervention (Day 6); primary follow-up (Day 11); and 4 weeks post-intervention (Day 33)], to assess the quality-of-life impact of MS (87), and undiagnosed sleep disorders (88), which might impact fatigue. Both scales will be analyzed as change-from baseline comparison between groups at the main clinical follow-up. Daily sleep quality ratings, integrated into the fatigue diary, will be analyzed analogously to diary-based fatigue scores.

#### Clinical tests and cognition

At baseline, participants will be assessed for their level of clinical disability, measured as the Extended Disability Status Scale (EDSS) (55) score. At baseline and follow-up, participants will complete the oral version of the Brief International Cognitive Assessment for Multiple Sclerosis (BICAMS) (89), comprising the California Verbal Learning Test (CVLT, recall 1-5) (90, 91), the revised version of the Brief Visuospatial Memory Test (BVMT-R) (92), and the Symbol Digit Modalities Test (SDMT) (93, 94). A modified Multiple Sclerosis Functional Composite (MSFC) (95), will also be administered, including the Timed 25-Foot Walk Test (T25FW) and 9-hole Peg Test (9HPT). The Paced Auditory Serial Addition Test (PASAT) will not be included due to overlap with SDMT, which demonstrates superior reliability (96, 97). All outcomes, SDMT, CVLT, BVMT-R, T25FW, and 9HPT, will be analyzed as between-group difference in change from baseline to main clinical follow-up.

#### Accelerometer-based outcomes

Participants will wear a wrist-mounted tri-axial accelerometer (wGT3X-BT, ActiGraph LLC, Pensacola, FL, United States) continuously from baseline until follow-up, including the intervention week. A meta-analysis of 21 studies with 1,098 subjects showed that people with MS are more sedentary and take fewer daily steps per day than healthy controls under free-living conditions (98). Higher daily physical activity in MS has been associated with lower fatigue levels (99, 100), independently of depressive symptoms (99). Given the sensitivity of wearable data to filtering and processing methods (101, 102), an exploratory analytic approach will be used to assess between-group differences.

#### Single-session outcomes of neurophysiology

All participants will undergo a neurophysiological assessment using TMS targeting left motor cortex immediately before and after the first intervention (D1). These measurements will evaluate cortical excitation-inhibition dynamics and serve both as exploratory biomarkers of fatigue mechanisms in people with MS and as indicators of functional target engagement induced by the paired-pulse low-frequency rTMS protocol. For acquisition specifics, we refer to the dedicated TMS-EMG section.

Single-pulse TMS will be used to measure the resting motor threshold (RMT), defined as the lowest stimulation intensity eliciting a motor evoked potential (MEP) of >50 μV in 5 out of 10 consecutive stimulations (103) and obtain input–output curves of mean MEP amplitudes at rest and during low-level muscle contraction (58). EMG recordings during contraction will used to derive the cortical silent period (CSP) in the contralateral and ipsilateral silent period in the ipsilateral hand (104). Paired-pulse TMS will evaluate short-interval intracortical inhibition (SICI) and intracortical facilitation (ICF) using established protocols, with 20 trials per condition to ensure reliability (58, 104).

#### Neuroimaging—structural and functional connectivity at ultra-high field (7 tesla)

Participants will undergo three MRI sessions using a 7T Philips Achieva scanner (Philips, Best, The Netherlands) at baseline, immediately after the final intervention (Day 5), and at primary follow-up (Day 11). For specific details on MR acquisitions, see later section.

At each timepoint, we will acquire ^1^H-MRS, for measures of GABA and Glu, diffusion-weighted imaging (DWI) for white matter microstructure and structural connectivity, and resting-state fMRI for functional connectivity analysis. Group comparisons will follow an exploratory longitudinal framework. In addition, structural whole-brain MRI images will be collected once across all the 3 scanning sessions for quantification of lesion load; tissue properties; and volumetric analyses. This includes MP2RAGE and 3D-T2 (on D0), 3D-FLAIR and multi-echo gradient echo scans (on Day 5), and double inversion recovery, magnetization transfer and multi-echo GraSE scans (on Day 11).

Missed sequences may be deferred to subsequent sessions or omitted entirely. Imaging data will contribute to exploratory analyses of fatigue-related neurobiology in MS.

#### Simulated E-fields

In addition to being an integral part of the intervention targeting algorithm, SimNIBS 4.5 allows for post-hoc simulation of the induced e-field for a given stimulation site. This allows important quality control for the *de facto* administered dose. The actual delivered electric field will be used in exploratory analyses of predictors of treatment effect.

#### Feasibility, side effects and adverse event monitoring

After every intervention session, participants will be asked to fill out the TMS Adverse Events and Associated Sensations Questionnaire (TMSENS_Q) (105), section 4 (and section 5 only in case of serious adverse events). This detailed questionnaire on adverse effects and sensations after TMS will provide detailed information on side effects to aid future decisionmakers. Feasibility will be assessed by drop-out rates between groups. Additionally, feasibility will be assessed by participants who complete follow-up through a semi-structured interview. This semi-structured interview is focused on the individual participant cost–benefit trade-off of effect against side effects of participation.

### Participant timeline

#### Trial overview

**Figure 1:**
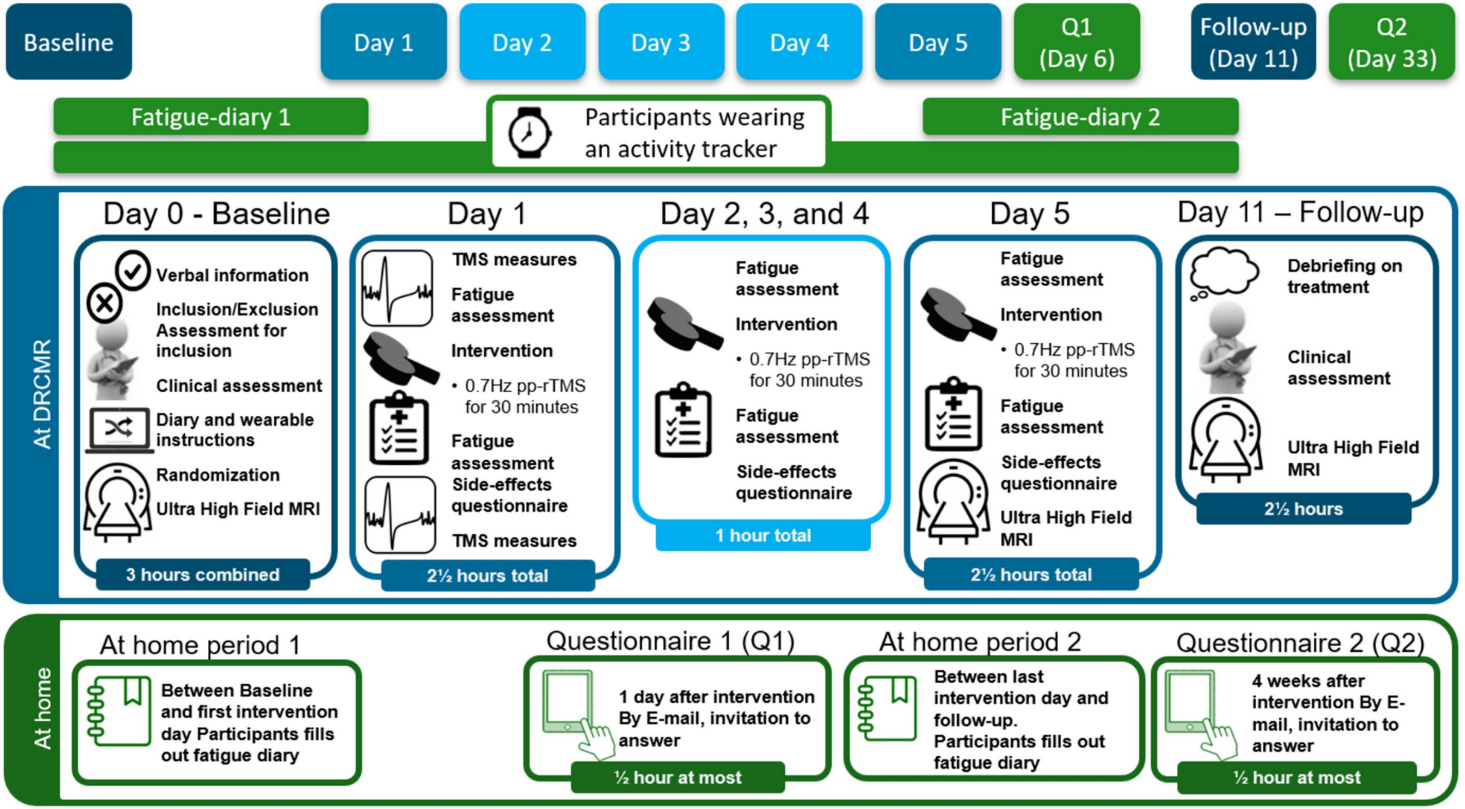
Study overview, showing contents of each experimental day. TMS, transcranial magnetic stimulation; pp-rTMS, paired-pulse repetitive; MRI, magnetic resonance imaging; Q1, 1st at-home questionnaire; Q2, 2nd at-home questionnaire; DRCMR, Danish research center for magnetic resonance.

**Figure 2:**
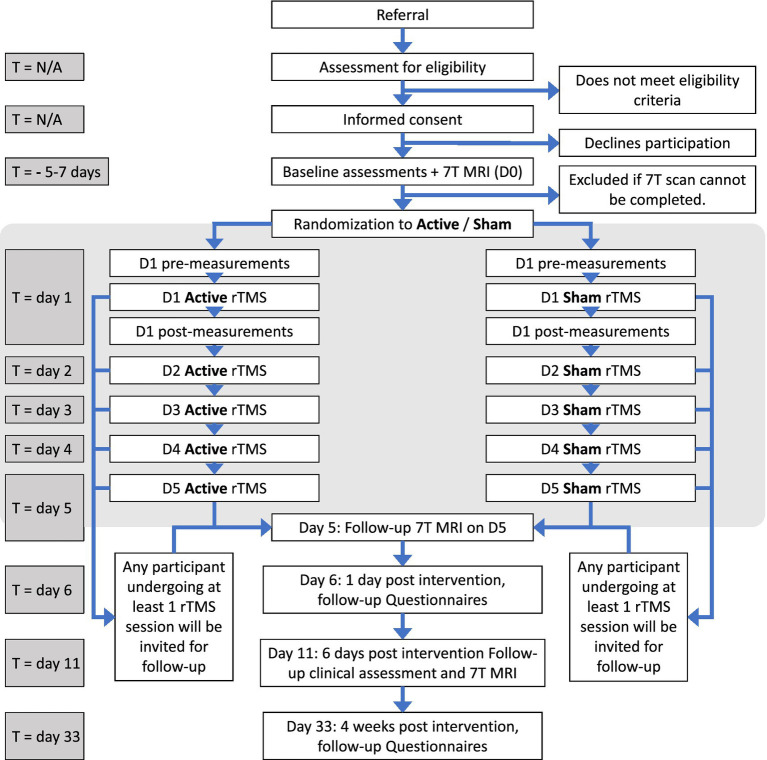
Timeline for participants. rTMS, Repetitive transcranial magnetic stimulation; MRI, magnetic resonance imaging.

The trial consists of a baseline period; an intervention period; and a follow-up period. See Figure 1 for graphical synopsis of the study protocol, see Figure 2 for a flowchart for participants. See Supplementary Table S1 for an overview of when specific data are acquired and assessments are performed.

All participants will perform the baseline visit immediately after enrolment or at the latest within 1 month of inclusion. The baseline visit (D0) comprises clinical testing, multimodal 7T MR scanning to acquire baseline neuro-metabolite concentrations (MRS); functional connectivity (rs-fMRI) and structural connectivity (DWI). The baseline visit is performed in the week prior to the intervention week, at least 5 days before the first intervention day. Between D0 and the first intervention day, participants will wear a wrist-worn accelerometer and fill out a fatigue diary.

The intervention spans five consecutive weekdays. On the first intervention day (Day 1), additional TMS-based measures of cortical excitation-inhibition balance will be conducted before and immediately after the first rTMS intervention. On the last intervention day (Day 5), participants will immediately undergo post-intervention 7T MRI, including MRS, rs-fMRI and DWI The primary follow-up will be scheduled on Day 11, 6 days after the last rTMS session, and includes clinical assessments and the third 7T MRI scanning session with MRS, rs-fMRI and DWI. The timing of the follow-up visit, at 6 days post intervention, was decided for logistical reasons, and fall well within previously reported therapeutical after effects of neuromodulation in fatigue in MS (50, 106) and depression (107). Between Day 5 and Day 11, participants will continue wearing a wrist-worn accelerometer and complete a fatigue diary. Additionally, participants will be asked to complete electronic patient-reported outcome measures 1 day post-intervention (Day 6) and again 4 weeks after the final session (Day 33).

### Sample size calculation

Based on a clinical meaningful effect size of 10 points on the FSMC scale between baseline (D0) and the primary follow-up (Day 11) measurements, and assuming a standard deviation of 12.5 (20, 21, 108), the estimated sample size is 25 per arm and 50 participants in total. This will yield 80 % power of reaching evidence of a treatment effect, with a *p*-value under 0.05. Accounting for a drop-out of 15%, our final sample size is 29 per arm, 58 in total.

### Recruitment procedure

Eligible patients will be identified by clinicians at the outpatient clinic at the Danish Multiple Sclerosis Centre, Copenhagen University Hospital - Rigshospitalet Glostrup. They will be provided with written information at least 24 h prior to enrolment and oral information about the study purpose and methods, prior to inclusion. To facilitate recruitment, there will be regular meetings across the 2 centers, and promotional talks to clinical (non-research) staff at the recruiting site. Additionally, flyers and recruitment material will be available for sharing across patient-networks and via the Danish Multiple Sclerosis Society (Scleroseforeningen).

## Methods: assignment of intervention

### Allocation and sequence generation

Participants will be randomly assigned (1:1) to the intervention or sham arm using a computer-generated sequence via block-randomization with variable block sizes (2, 4, or 6 participants) to ensure balanced group sizes and minimize allocation predictability. This approach controls for potential bias related to evolving staff expertise over time. The sequence is generated using MatLab (The MathWorks Inc., Natick, MA, United States) with a length of at least 70 to account for potential dropouts. Randomization was performed by author VW, who is not involved in intervention delivery or outcome assessment. All other personnel are blinded to the treatment status.

### Allocation concealment and implementation

Allocation concealment will be maintained using sealed, opaque envelopes opened only on the first intervention day. Each participant receives a unique 6-digit code which will be distinct from the subject ID used for data management. The code will be entered into the stimulator prior to each session and does not disclose group allocation. The randomization key, containing block order and treatment assignment, will be securely stored and inaccessible to investigators involved in intervention delivery until trial completion.

Participants will be recruited and enrolled by an investigator not involved in sequence generating and envelope preparation. Participants will have completed all baseline behavioral and 7T MRI assessments prior to group assignment. Envelopes will be opened before baseline TMS measurements, on the first intervention day.

### Blinding

Allocation blinding is maintained through the integrated design of the coil and stimulator. The coil provides no visual cues regarding its active or sham side and automatically detects its orientation via an internal sensor. The sham side is engineered to replicate the auditory and tactile peripheral co-stimulation created by active stimulation. The stimulator displays no indication of condition assignment. To preserve blinding between consecutive participants, the coil is removed and reattached for each session.

To mimic the cutaneous sensation associated with active TMS pulses and preserve blinding, all participants, regardless of group allocation, will receive low-intensity electrical stimulation to the forehead, positioned as close as possible to the stimulation site while respecting the individual’s hairline. This electrical stimulation is a built-in feature of the B65-Cool-A/P coil and MagPro XP Orange Edition stimulator.

The electrical stimulation intensity will be automatically adjusted by the stimulator to match the sensation produced by the magnetic coil, using a fixed scaling factor. This factor will be applied uniformly across all participants to standardize the sensory experience and minimize the risk of unblinding for both participants and intervention administrators. Internal pilot testing with TMS-experienced individuals confirmed that this setup provides a credible sham condition. Success of blinding will be assessed by asking each participant to indicate which group allocation they received, as part of the TMSens questionnaire (105).

Emergency unblinding requests will be evaluated individually. If necessary, unblinding will involve simulating a session and manually verifying the treatment condition based on device output.

## Methods: data collection, management and analysis

### Data collection methods and management

#### Data collection

##### Questionnaires

Questionnaire data are collected and managed using REDCap (Vanderbilt University, TN, United States), a secure, web-based software platform designed to support data capture for research studies, hosted at the Capital Region of Denmark (RegionH) (109, 110). Access to REDCap is granted through an electronic tablet (Apple, California, United States) for visits, and by an electronic link to the participants’ emails for at-home questionnaires.

##### MRI/MRS data acquisition

Across the three scan sessions, the scans take place in the same order: Scouts + calibration scans; MPRAGE for MRS planning; MRS; Structural 1; rs-fMRI; DTI; other structural scans. The specific aim is to always have equal gradient history for the MRS, as this may affect and bias the results (111). All MRI scans are quality-checked immediately after acquisition by trained staff and repeated if of poor quality. All imaging parameters are detailed in Supplementary Table S2.

^1^H-MRS will be acquired with a semi-localized by adiabatic selective refocusing (sLASER) sequence with a voxel 20 mm cubic, as in (112). Voxel placement will be done on a T1-weighted MPRAGE image, acquired immediately prior to the sLASER scan. MRS is performed at 3 voxel positions per session: Bilateral M1 hand knob (113), covering also the PMd; and a midline parietal voxel, corresponding to the inferior portion of the precuneus. Voxel placement will be aided by SmartBrain™ (Philips, Best, Netherlands) and manually corrected.

##### TMS

Single and paired-pulse TMS is performed pre- and post-intervention on intervention day one to capture changes in motor cortex excitation and inhibition. Experiment parameters are in accordance with safety guidelines (58). The cortical motor hotspot is defined as the corresponding scalp position at which TMS induces the largest and most consistent MEP in the first dorsal interosseus muscle (FDI). The location will be recorded and maintained using a stereotactic neuronavigation system for coil placement and continued control of correct coil position (Localite, Bonn, Germany) (114).

TMS stimulations will be delivered through a handheld B65 coil attached to a MagVenture X100 MagOption stimulator (MagVenture, Farum, Denmark). TMS-evoked EMG potentials are recorded with self-adhesive surface electrodes (Neuroline, Ambu, Ballerup, Denmark) attached to the right and left FDI muscle using a belly-to-tendon montage, with ground on right side ulnar styloid process. Electromyographic (EMG) signals will be sampled at 5 kHz, band-pass filtered (2–2,000 Hz) and amplified (500 times) using an eight-channel DC amplifier (D360, Digitimer, Hertfordshire, United Kingdom), digitized (Micro 1401-4 w/ ADC12, Cambridge Electronic Design, Cambridge, United Kingdom), and stored and displayed using Signal software version 8.0 (Cambridge Electronic Design, Cambridge, United Kingdom).

TMS pulses will be given every 4 s with 20% jitter when evoking MEPs in the relaxed target muscle. TMS during pre-contraction will use a lower ISI (3 s) to avoid fatigue. We will perform at least 15 trials per condition for recruitment-curves; 20 for paired-pulse experiments; and 21 (across bouts of 7, to avoid further fatigue) for ipsilateral silent period. More trials will be done if online data quality assessment deems this required for proper data quality. F- and M-waves will be evoked by supramaximal electrical stimulation of the right ulnar nerve (Digitimer DS7A, Digitimer, Cambridge, UK), their onsets latencies will be used to compute peripheral and central motor conduction times (104).

#### Data storage and management

All data entered will be kept in the electronical case report form REDCap and all MRI, EMG and accelerometer data will be stored on a secure server owned and operated by the DRCMR. Servers are regularly backed up by the IT-department of the Capital Region of Denmark, ensuring data integrity. All data, including data entered on REDCap, TMS and MRI data are reviewed during each session for quality and completeness. All data will be pre-processed between sessions. Missing data will be addressed at the last study visit. Data access will be limited to the study coordinator and sub-investigators involved in the study.

### Training of staff

#### Training prior to performing MRI experiments

All personnel involved in MRI procedures will have completed the department’s 7T-specific “MRI driver’s license” program, which includes comprehensive safety training according to current guidelines (115). During all scans, a medical doctor and a physicist will be available for assistance, if needed. There will always be at least two trained staff members present during the MRI scanning sessions.

#### Training prior to performing TMS experiments

All personnel involved in TMS and rTMS procedures will have completed the department’s “TMS driver’s license” program, which includes comprehensive safety training and supervised protocol-specific TMS sessions (41, 42). A dedicated TMS researcher with extensive expertise in EMG-based neurophysiology will be present for all Day 1 assessments to ensure high-quality data acquisition. All TMS and rTMS sessions will be conducted in the presence of a minimum of two trained staff members.

#### Statistical methods and analysis

All variables will be visually inspected for normal distribution through histograms and quantile-quantile plots prior to analysis and appropriately transformed if necessary. If data diverge from the normal distribution after transformation non-parametric testing will be performed. All tests will be 2-sided and statistical significance will be set at a *p*-value < 0.05. Data will be analyzed with R (116), Matlab (The MathWorks Inc., Natick, MA, United States) or similar software.

All data will be analyzed as a modified intention-to-treat, where all participants who have undergone at least one rTMS session (active or sham) and have follow-up data for outcomes will be analyzed according to the group they were randomized to. No per-protocol analyses are planned. The primary outcome will be analyzed as difference in change between baseline and clinical follow-up, between active and sham group. The statistical significance will be assessed with a mixed-effects linear model with the baseline as the reference. The primary analysis model will not adjust for depression, sleep, or physical activity measures, as the study is powered to detect overall treatment effects rather than covariate-adjusted effects. These variables will be examined in exploratory analyses as potential modifiers of treatment response, to assess whether treatment effects differ according to baseline levels or changes in these factors. Secondary outcomes with two timepoints (baseline and follow-up), will be compared as the difference in change between groups. Secondary outcomes with more timepoints will be analyzed with mixed-effects linear models with the baseline as reference. Neither the pre-registered primary or main secondary outcomes will be corrected for multiple comparisons, except where appropriate due to multiple measurements per participant. Exploratory analyses will be appropriately corrected for multiple comparisons prior to reporting.

There are no plans for predetermined subgroup analyses.

### Methods: monitoring

#### Data monitoring

Confidential documents will be stored in a locked file, while the electronic information that can be traced to an identifiable person will be stored on a password-protected computer behind a secure “firewall” in accordance with the Danish Privacy Act.

#### Adverse events monitoring and harms

Minor discomfort due to peripheral co-activation from the rTMS treatment, e.g., scalp sensations; discomfort from peripheral electrical stimulation. Minor discomfort will be assessed with the TMS-Sens-Questionnaire (105), as described elsewhere in this protocol. Additionally, slight discomfort related to 7T MRI (see safety section) is expected.

All serious adverse events, or unexpected side effects will be reported to relevant authorities including the sponsor and the Danish Medicines Agency (in Danish: Lægemiddelstyrelsen). All adverse events will be recorded in the electronic clinical report form.

##### Safety

The utilization of non-invasive brain stimulation, in the form of TMS and rTMS, as well as 7T MRI, lead to specific safety concerns that require proper screening. TMS and rTMS are generally considered safe, according to latest guidelines (42), meta-analysis (117), and have also been shown safe in MS (45). Additionally, we will screen for adverse sensations using the TMSens questionnaire (105). 7T MRI is generally considered safe with proper precautions (56, 118), with few and not clinically relevant physiological side effects (119–121), no cognitive side effects (122), and is generally tolerated (122–124), including at our facility, in MS participants (112, 125).

##### Post-trial or ancillary care, in case of harm

All participants are covered by the general national insurance of the healthcare sector of Denmark (in Danish: Patientforsikringen).

#### Plans for auditing

The study is monitored within the department by members outside of the research project team. The medical ethics board may audit study documents at any time.

### Ethics and dissemination

#### Research ethics approval

This study has been approved by the national Medical Research Ethics Committee (in Danish: *Videnskabsetisk Medicinsk Komité*) under the Danish Health Authority (in Danish: *Sundhedsstyrelsen*) in September 2023 (ID 2309733), according to the Declaration of Helsinki. Ethics approval was amended in May 2024 and June 2025. Approval has also been granted by the Danish Data Protection Agency (ID: p-2023-14641). The study is pre-registered at ClinicalTrials.gov (ClinicalTrials.gov, ID: NCT06569550).

#### Protocol amendments

A protocol amendment was added after the initial piloting phase, before inclusion of any participant, in May 2024. It was implemented based on patient representative feedback and feasibility. Because it was implemented prior to recruitment of the first participant, it will not be discussed further. A second protocol amendment was approved on June 6th, 2025, which altered the inclusion criteria but did not alter the intervention or outcomes of interest. Specifically, the criteria were widened as follows: The upper age-limit for inclusion was increased from 55 to 65; secondary progressive MS participants can now be included; the intake of SSRIs and SNRIs are no grounds for exclusion. Any further protocol amendments will be communicated to ClinicalTrials.gov as well as collaborators.

The SPIRIT reporting guidelines were used for reporting the contents of this study protocol (126).

#### Consent

Patients will be informed about the study and its contents by both oral and written material. Participants are given up to 24 h to consider participation. Informed consent is obtained, in writing, by a trained study coordinator. There is no post-trial care, after the last follow-up. Nor is there any anticipated harm or compensation for trial participation.

#### Confidentiality

To ensure confidentiality, all participants are assigned a random and unique study ID. This study ID will only co-occur with their real identity on one form, filled out at inclusion, which will be kept separate from the dataset. Participant names will never be included in the dataset.

#### Dissemination policy

Positive, negative and null results will be published in peer-reviewed international journals as well as be presented at national and international conferences. Results will be published adhering to the CONSORT guidelines (127, 128). Co-authorship will comply with the ICMJE recommendations (Vancouver guidelines). Once concluded, a letter will be offered to all participants explaining the results of the study in lay language.

In accordance with Danish data protection legislation, it is currently not allowed to publish individual participant data. Data that underlie published results related to the primary and secondary outcome measures will, following anonymization, be made available upon reasonable request. However, data will be grouped in appropriate categories with minimum 5 participants in each group.

## Discussion

Fatigue is one of the most prevalent and disabling symptoms in MS, yet effective treatment options remain lacking (24–26). This study protocol investigates a novel, biologically informed rTMS intervention targeting the left PMd, a region implicated in the pathophysiology of MS-related fatigue. The trial is sufficiently powered and designed to provide mechanistic insights alongside clinical outcome data.

To the best of our knowledge, only one published clinical trial has targeted fatigue as a primary outcome using rTMS in MS (45). That study employed high-frequency stimulation of M1 or left PFC with non-focal H-coils (129), was primarily a safety study and found no significant therapeutic effect. Other trials assessing fatigue as a secondary outcome show considerable heterogeneity in stimulation parameters and cortical targets (46–49). Notably, two studies reported superiority of rTMS over placebo treatment for fatigue alleviation (46, 48), although neither had fatigue as the primary outcome, and neither employed protocols with mechanistic grounding in premotor network dysfunction.

The rationale for PMd targeting is supported by both functional and anatomical considerations: Neurophysiological studies in healthy participants have shown reduced corticospinal excitability in left M1-HAND after 30 min of low-frequency rTMS of left PMd (34, 35). Previous studies have found TMS to have both local and remote effects within the targeted network, when targeting the left PMd (130–132). Accordingly, the low-frequency paired-pulse rTMS protocol that will be used in this trial suppresses cortical activity locally in the PMd as well as in connected motor regions (40). Functionally, the premotor network acts as a sensorimotor integration hub (131, 133, 134), and its dysfunction has been repeatedly implicated in MS-related fatigue (20, 21, 29). Anatomically, the PMd is readily accessible to TMS due to its superficial location (133), allowing for effective stimulation with lower intensities and reduced off-target current spread compared to deeper or less focal targets (129, 135). Together, these functional and anatomical considerations motivate the use of this novel paired-pulse rTMS protocol to target the PMd for modulating excitation–inhibition balance in a targeted and network-specific manner.

Our study incorporates a multidimensional biomarker strategy using 7T ^1^H-MRS to measure regional glutamate and GABA concentrations. Additionally, we will assess cortical excitation–inhibition dynamics using TMS-EMG before and after a single intervention, positioning these measures as candidate indicators of both treatment engagement and underlying mechanisms of fatigue.

Recognizing the link between physical activity and fatigue improvement (27, 29), we quantify real-world activity using a wrist-worn accelerometer and assess objective fatigability via sustained grip force measurements (73), complementing patient-reported fatigue outcomes. Feasibility and tolerability of the novel paired-pulse rTMS intervention over five consecutive days will also be systematically evaluated through dropout rates, adverse event tracking, and post-treatment patient feedback, including whether participants would recommend rTMS to other persons with MS.

By integrating mechanistic, behavioral, and feasibility endpoints, this trial aims to clarify the role of premotor network dysfunction and cortical excitation–inhibition imbalance in MS-related fatigue. The findings will inform both future clinical trials and the development of targeted neuromodulatory interventions in MS symptom management.

## Footnotes

1
https://simnibs.github.io/simnibs
