# Supplementary material for: Repetitive transcranial magnetic stimulation to alleviate fatigue in multiple sclerosis—study protocol for a randomized sham-controlled double-blinded clinical trial
Source: Front Neurol. 2026 Feb 26;17:1759679. doi: 10.3389/fneur.2026.1759679 (PMC12979102; doi:10.3389/fneur.2026.1759679)
Supplement: Supplementary file 1 [file Table_1.docx]

# Supplementary material

This supplementary material contains 2 tables.

**Supplementary table 1, overview of measurements done as part of study:**

| TABLE 1 | | | | | | | | | | | | |
| --- | --- | --- | --- | --- | --- | --- | --- | --- | --- | --- | --- | --- |
| Activity/Assessment | Pre-study | Base-line | Pre-week | Intervention week | | | | | Post-week | Short term questionnaire | Follow-up D11 | Long term questionnaire |
| Timepoint |  | T=0 |  | D1 | D2 | D3 | D4 | D5 |  | +1 day | Day 11 | +4 weeks |
| Pre-screening | X |  |  |  | | | | |  |  |  |  |
| Consent form |  | X |  |  | | | | |  |  |  |  |
| Inclusion/Exclusion Criteria |  | X |  |  | | | | |  |  |  |  |
| Randomization |  | X |  |  | | | | |  |  |  |  |
| Demographics and clinical information:   - Age - Gender - Medication |  | X |  |  | | | | |  |  |  |  |
| Questionnaires | | | | | | | | | | | | |
| EHI |  | X |  |  | | | | |  |  |  |  |
| FSMC |  | X |  |  | | | | |  | X | X | X |
| MDI |  | X |  |  | | | | |  |  | X |  |
| MFIS |  | X |  |  | | | | |  |  | X |  |
| FSS |  | X |  |  | | | | |  |  | X |  |
| BDI-ii |  | X |  |  | | | | |  |  | X |  |
| PFS |  | X |  |  | | | | |  | X | X | X |
| ESS |  | X |  |  | | | | |  | X | X | X |
| MSIS-29 |  | X |  |  | | | | |  | X | X | X |
| VAFS |  |  |  | X | X | X | X | X |  |  |  |  |
| TMS-Sens-Q (baseline demographics) |  | X |  |  | | | | |  |  |  |  |
| TMS-Sens-Q (session specific info) |  |  |  | X | X | X | X | X |  |  |  |  |
| TMS-Sens-Q (side effects) |  |  |  | X | X | X | X | X |  |  |  |  |
| Clinical tests | | | | | | | | | | | | |
| EDSS |  | X |  |  | | | | |  |  |  |  |
| 9HPT |  | X |  |  | | | | |  |  | X |  |
| T25FW |  | X |  |  | | | | |  |  | X |  |
| SDMT |  | X |  |  | | | | |  |  | X |  |
| CVLT1-5 |  | X |  |  | | | | |  |  | X |  |
| BVMT-R |  | X |  |  | | | | |  |  | X |  |
| Fatiguability Index |  | X |  |  | | | | |  |  | X |  |
| MR measures | | | | | | | | | | | | |
| T1w |  | X |  |  |  |  |  | X |  |  | X |  |
| MP2RAGE |  | X |  |  | | | | |  |  |  |  |
| T2w TSE |  | X |  |  | | | | |  |  |  |  |
| FLAIR |  |  |  |  |  |  |  | X |  |  |  |  |
| SWI |  |  |  |  |  |  |  | X |  |  |  |  |
| DIR |  |  |  |  | | | | |  |  | X |  |
| MTR |  |  |  |  | | | | |  |  | X |  |
| MEGRASE |  |  |  |  | | | | |  |  | X |  |
| DWI |  | X |  |  |  |  |  | X |  |  | X |  |
| MRS M1/PMd ipsilateral |  | X |  |  |  |  |  | X |  |  | X |  |
| MRS M1 /PMd contralateral |  | X |  |  |  |  |  | X |  |  | X |  |
| MRS parietal control |  | X |  |  |  |  |  | X |  |  | X |  |
| Rs-fMRI |  | X |  |  |  |  |  | X |  |  | X |  |
| At-Home measurements | | | | | | | | | | | | |
| Accelerometer wearing |  |  | X | X | X | X | X | X | X |  |  |  |
| Fatigue Diary fill-out |  |  | X |  | | | | | X |  |  |  |
| Neurophysiology/TMS | | | | | | | | | | | | |
| RMT |  |  |  | X |  |  |  |  |  |  |  |  |
| Recruitment curve rest |  |  |  | X |  |  |  |  |  |  |  |  |
| Recruitment curve activity |  |  |  | X |  |  |  |  |  |  |  |  |
| SICI+ICF |  |  |  | X |  |  |  |  |  |  |  |  |
| ISP |  |  |  | X |  |  |  |  |  |  |  |  |
| CSP |  |  |  | X |  |  |  |  |  |  |  |  |
| Intervention/Sham |  |  |  | X | X | X | X | X |  |  |  |  |

Legend: **EHI** Edinburgh Handedness Inventory; **FMSC** Fatigue Scale for Motor and Cognitive Symptoms; **MDI** Major Depression Inventory; **MFIS** Modified Fatigue Impact Scale; **FSS** Fatigue Severity Scale; **BDI-ii** Becks Depression Inventory, second edition; **PFS** Pittsburgh Fatigability Scale; **ESS** Epworth Sleepiness Index; **MSIS-29** Multiple Sclerosis Impact Scale, 29 items; **VAFS** Visual Analogue Fatigue Scale; **TMS-Sens-Q** Transcranial Magnetic Stimulation Adverse Sensations and Effects Questionnaire; **EDSS** Extended Disability Status Scale; **9HPT** 9-hole Peg Test; **T25FW** Times 25-foot Walk; **SDMT** Symbol Digit Modalities Test; **CVLT1-5** California Verbal Learning Test, immediate recall 1-5; **BVMT-R** Brief Visual Memory Test, revised; **MP2RAGE** Magnetization Prepared 2 Rapid Gradient Echo; **TSE** Turbo Spin Echo; **FLAIR** Fluid-Attenuated Inversion Recovery; **MEGRE** Multi-Echo Gradient Echo; **DIR** Double Inversion Recovery; **MTR** Magnetic Transfer Rate; **MEGRASE** Multi-Echo Gradient and Spin Echo; **DWI** Diffusion Weighted Imaging; **MRS** Magnetic Resonance Spectroscopy; **M1/PMd** Primary Motor Cortex/Dorsal Premotor Cortex; **rs-fMRI** Resting State Functional Magnetic Resonance Imaging; **TMS** Transcranial Magnetic Stimulation; **RMT** Resting Motor Threshold; **SICI** Short Intracortical Inhibition; **ICF** Intracortical Facilitation; **ISP** Ipsilateral Silent Period; **CSP** Cortical Silent Period.

**Supplementary Table 2, overview of MR scan parameters:**

| Table 2: | | | | | | | | |
| --- | --- | --- | --- | --- | --- | --- | --- | --- |
| Sequence | TE (ms) | TR (ms) | FOV | Voxelsize (actual/reconstructed) | Flip angle | Time (mm:ss) | SENSE (P:phase dir, S:slice dir) | Other |
| 3D-MPRAGE  (SAG) | 2.2 | 4.9 | 246x246x174mm | 1.00x1.00x1.00 mm  0.85x0.85x1.00 mm | 7 | 1:55 | 2/2.5 (P/S) | - |
| MRS | 32 | 3175 | - | 20x20x20 mm | 90 | 3:30 each | - | NSA 64; samples 2048, spectral BW 4000; |
| DTI  (TRA) | 54 | 13934 | 200x200x121mm | 1.79x1.85x1.80 mm  1.79x1.79x1.90 mm | 90 | 8:08 + 1:38 | 4 (P) | PA: 32 dir with b=1000, 1 b=0 volumes;  AP: 5 b=0 volumes |
| rs-fMRI  (TRA) | 23 | 1500 | 216x216x150mm | 2.00x2.00x2.00 mm,  1.93x1.93x2.00 mm | 65 | 0:57; 7:42 | 2.4 (P); MULTIBAND SENSE 3, shift 2 | 30 volumes PA; 300 with AP phase encoding. With cardiac and respiration monitoring |
| 3D- MP2RAGE  (SAG) | 2.5 | 6.8 | 224x224x180mm | 0.70x0.70x0.70 mm  0.58x0.58x0.70 mm | 5; 3 | 7:02 | 2/1 (P/S) | TI1/TI2 = 909/2759ms |
| 3D-T2-TSE  (SAG) | 313 | 3696 | 256x256x190mm | 0.80x0.80x1.60 mm  0.40x0.40x0.80 mm | 100 | 5:59 | 2/1 (P/S) | TSE factor 200 |
| 3D-FLAIR (SAG) | 291 | 7242 | 230x230x200mm | 0.70x0.70x1.00 mm,  0.69x0.69x1.00 mm | 90 | 11:06 | 2.2/2.2 (P/S) | IR = 2200 ms |
| 3D-ME-GRE (TRA) | 5 | 24 | 172x219x117mm | 0.65x0.64x0.70 mm; 0.28x0.29x0.70 mm | 14 | 11:12 | 2/1 (P/S) | 4 echoes; delta TE 5.2 ms |
| 3D-MTR (SAG) | 3.9 | 8 | 230x230x179mm | 0.90x0.90x1.80 mm  0.90x0.90x0.90 mm | 4 | 3:18 +; 1:20 | 1/1 (P/S) | Pulse: Sinc-Centre, Δf=2000Hz |
| 3D-DIR (SAG) | 299 | 9500 | 230x230x168mm | 0.99x1.00x2.00 mm 0.44x0.44x1.00 mm | 55 | 6:49 | 2/1.5 (P/S) | IR1/IR2= 3275/ 465 ms |
| 3D-ME-GRASE  (TRA) | 7 | 2000 | 239x199x158mm | 1.25x1.42x3.00 mm,  1.25x1.25x1.50 mm | 90 | 12:02 | 2.8/2.5 (P/S) | 32 echoes; delta TE 7 ms |

Legend: **TE** Echo Time; **TR** Repetition Time; **FOV** Field of View; **MP2RAGE** Magnetization Prepared 2 Rapid Gradient Echo; **TSE** Turbo Spin Echo; **FLAIR** Fluid-Attenuated Inversion Recovery; **ME** Multi-echo; **GRE** Gradient Echo; **DIR** Double Inversion Recovery; **MTR** Magnetization Transfer Ratio; **GRASE** Gradient and Spin Echo; **DWI** Diffusion Weighted Imaging; **MRS** Magnetic Resonance Spectroscopy; **rs-fMRI** Resting State Functional Magnetic Resonance Imaging; **IR** Inversion Recovery; **TI** Inversion Time; **SAG** sagittal slice orientation; **TRA** transverse slice orientation
